# Comparison of the Effectiveness of Various Drug Interventions to Prevent Etomidate-Induced Myoclonus: A Bayesian Network Meta-Analysis

**DOI:** 10.3389/fmed.2022.799156

**Published:** 2022-04-26

**Authors:** Kang-Da Zhang, Lin-Yu Wang, Dan-Xu Zhang, Zhi-Hua Zhang, Huan-Liang Wang

**Affiliations:** ^1^Department of Anesthesiology, Qilu Hospital of Shandong University, Jinan, China; ^2^Shenzhen Research Institute of Shandong University, Shenzhen, China

**Keywords:** etomidate, myoclonus, anesthesia induction, network meta-analysis, Bayesian framework

## Abstract

**Background:**

Myoclonic movement is a very common but undesirable phenomenon during the induction of general anesthesia using etomidate. Such movement may cause unnecessary problems. Currently, there is an increasing number of drugs for preventing etomidate-induced myoclonus (EM). However, direct comparisons of various drugs are lacking, and this interferes with clinical decision-making. Our network meta-analysis (NMA) aimed to compare the efficacy of different drugs for the prevention of moderate-to-severe general myoclonus.

**Methods:**

Using several biomedical databases, randomized controlled trials (RCTs) published in English from inception to August 22, 2021 were searched. Among the various interventions, we selected nine types of intervention drugs (dexmedetomidine, etomidate, lidocaine, NMDA receptor antagonist, κ opioid receptor agonist, μ opioid receptor agonist, muscle relaxant, gabapentin, and midazolam) for comparison, according to the number of studies. Bayesian NMA was performed using STATA16 and R softwares. The relative risk of EM was assessed using risk ratios (RRs) and the corresponding 95% confidence intervals (CI).

**Results:**

A total of 31 RCTs (3209 patients) were included. NMA results showed that, compared with a placebo, etomidate (RR 4.0, 95%CI 2.1–7.8), κ opioid receptor agonist (RR 2.9, 95%CI 1.9–4.6), μ opioid receptor agonist (RR 3.1, 95%CI 2.3–4.3), NMDA receptor antagonist (RR 1.7, 95%CI 1.0–2.8), dexmedetomidine (RR 2.4, 95%CI 1.5–3.9), lidocaine (RR 2.1, 95%CI 1.2–3.9), and midazolam (RR 2.2, 95%CI 1.5–3.2) can significantly reduce the risk of EM. In contrast, the effects of muscle relaxants (RR 2.1, 95%CI 0.81–5.3) and gabapentin (RR 2.8, 95%CI 0.92–9.3) were inconclusive. Further subgroup analyses showed that preoperative low-dose etomidate, μ-opioid receptor agonist, and κ-opioid receptor agonist were significantly better than other interventions in the prevention of moderate to severe EM.

**Conclusion:**

Preoperative use of small doses of etomidate or opioids may be the most effective way to avoid EM, especially moderate and severe EM, which makes anesthesia induction safer, more stable, and aligns better with the requirements of comfortable medicine.

**Systematic Review Registration:**

[https://www.crd.york.ac.uk/prospero/], [CRD4202127706].

## Introduction

Etomidate, a compound containing an imidazole carboxyl group, was introduced to clinical practice in the 1970s. In addition to its strong anesthetic efficacy, rapid onset, and rapid recovery, etomidate has the advantages of stable cardiovascular profiles and minimal respiratory depression, making it an ideal substitute for propofol and the first-line anesthetic for many elderly people and patients with impaired hemodynamics and cardiac reserve (1, 2).

However, etomidate often induces spontaneous movements, especially myoclonic activities. Generalized convulsive seizures may occur in severe cases, with an incidence of 50%–80% (3). Myoclonus is also related to epileptiform activities. Therefore, epileptic activities may be enhanced in the EEG of some patients after etomidate injection (4). According to the classical definition, myoclonus is a sudden, brief, lightning-like muscle jerk arising from an abnormality of the nervous system, excluding short or prolonged movements caused by the muscle itself such as fasciculation, spasms, or cramps (5). Myoclonus can damage muscle fibers and cause serum potassium to rise. Transient mild myoclonus may not be pathologically significant in most patients, but severe myoclonus can have unintended consequences, especially in patients with a full stomach, malignant hypertension, open eye injury, aneurysms, and hyperkalemia (6, 7).

In the past few decades, many drugs have been used in clinical practice for the prevention and treatment of EM, including opioids, benzodiazepines, dexmedetomidine, ketamine, lidocaine, magnesium sulfate, muscle relaxants, antiepileptics, and preoperative low-dose etomidate. The variety of drugs available is appreciated by many anesthesiologists. Some traditional pairwise meta-analyses have evaluated the efficacy of two drugs or a drug versus a placebo to guide agent selection (8–15). However, when faced with a wide range of interventions, most anesthesiologists still struggle to choose the best option, and instead, use the drug empirically. Furthermore, traditional meta-analyses cannot clearly rank different classes of interventions based on their efficacy outcomes.

Owing to the limitations of standard pairwise meta-analyses, we adopted a network meta-analysis (NMA) to determine the most effective approach for preventing myoclonus. NMA integrates direct and indirect evidence and enables the evaluation of multiple treatments in a single analysis (16). In this study, we determined the effectiveness of all interventions, as well as their ranking probabilities in overall and subgroup networks by summarizing the available evidence. The results of this study will provide evidence for the best preventive measure of moderate to severe myoclonus, when using etomidate.

## Methods

### Protocol and Registration

This systematic review followed the recommendations of the Preferred Reporting Items for Systematic Reviews and Meta-Analyses (PRISMA) guidelines (17), and was registered under the PROSPERO International prospective register of systematic reviews on October 6, 2021 (registration number CRD42021277063).

### Search Strategy

The search strategy was first designed jointly by the two authors, and then, the search was conducted independently. PubMed, Embase, the Cochrane Central Register of Controlled Trials (CENTRAL), and NIH ClinicalTrials.gov were searched to find relevant articles from inception to August 2021 within the restriction limit of “randomized controlled trial” and “English-language.” Some of the English literature from the CNKI database was supplemented. Using the combination of MeSH medical subject words and item words, the search terms were combined for literature retrieval through the logical characters “OR” and “AND.” Relevant search strategies are provided in the Supplementary Material.

All citations were downloaded and imported into EndNote for management (18). First, duplicates were excluded from the analysis. The titles and abstracts were then reviewed, and studies that did not meet the inclusion criteria were excluded. Finally, the full text of any potential study was analyzed and further screened according to the exclusion criteria. The above tasks were also performed by two authors independently. The reasons for article exclusion were recorded for the preparation of the literature screening flowchart.

### Inclusion and Exclusion Criteria

Inclusion criteria were formulated according to the PICOS framework (19), as follows: (1) adult patients who are purposed to surgical or invasive intervention under etomidate; (2) interventions including opioids, lidocaine, ketamine, dexmedetomidine, etomidate, muscle relaxant, magnesium sulfate, gabapentin, and midazolam; and (3) the control group could be a placebo or a comparison between the above drugs; (4) the outcome was the incidence of myoclonus induced by etomidate, and the degree of myoclonus was divided into none, mild (mild myoclonus in the face and/or upper limbs and/or distal lower limbs), moderate (some movement in the face and/or limbs), and severe (movement in limbs and trunk); and (5) the study must be a randomized controlled trial and published in English.

Studies were excluded if they included the following characteristics: (1) patients who had neuropsychological disease; adrenal cortex dysfunction; heart failure; renal, pulmonary, hepatic, or endocrine diseases; history of allergic reaction to etomidate and other study drugs; (2) patients who had taken sedative and analgesic drugs on the day of operation; and (3) lack of necessary outcomes to be extracted, for example, incomplete data.

### Data Extraction and Methodological Quality Assessment

We created a unified information extraction table in advance. Two authors independently screened the information, and any discrepancies were resolved through discussion. The following information was extracted: author’s name, publication year, age distribution, type of surgery, American Society of Anesthesiologists (ASA) physical status, induction dose of etomidate, treatment, sample size, and outcome. The primary outcomes were the incidence of EM and moderate-to-severe EM.

For randomized controlled trials, two reviewers independently applied the Cochrane Review Manager (Version 5.4) to assess the risk of bias (ROB) in randomized trials (20). The Cochrane Collaboration’s bias risk assessment tools are well-structured and mainly included random sequence generation, allocation concealment, blinding of participants and personnel, blinding of outcome assessment, incomplete outcome data, selective reporting, and other biases. Each trial was independently performed by two reviewers and classified as low-, unclear-, or high-risk. The Grades of Recommendation, Assessment, Development, and Evaluation (GRADE) Working Group recommended a four-step evidence quality grading for network meta-analyses (21). The certainty of the evidence was appraised as high, moderate, low, or very low.

### Statistical Analysis

We first constructed a network evidence plot using Stata16.0, and conducted a traditional pairwise direct comparison meta-analysis. The network plot clearly showed whether there was a direct comparison, and whether the effect between interventions was the result of direct comparison, indirect comparison, or a combination of the two. Heterogeneity was assessed between studies using the Q test and I^2^ statistic (22). If the P value of Cochran’s Q test statistic was less than 0.05, or the I^2^ statistic was greater than 50%, large heterogeneity between studies was determined, and the random-effects model was preferred. A pairwise meta-analysis was performed using the random-effects model. For binary outcomes, we reported the risk ratios (RR) and corresponding 95% confidence intervals (CI). If the 95%CI did not include 1, the difference between the two comparisons was considered statistically significant. A comparison-adjusted funnel plot was used to determine the possibility of a publication bias.

Owing to the existence of closed rings in the network evidence graph, we used a Bayesian network meta-analysis to compare the differences between different interventions (23). The “*gemtc*” package and the “*rjags*” package of R software that invoke the JAGS software^1^ for NMA based on a Bayesian generalized linear model were used (24, 25). For each outcome, the fixed-effects model and random-effects model were used for evaluation. The fitting degree of the model was determined by the deviance information criterion (DIC), and a model with less DIC was generally selected (26). Four Markov chains were used to set the initial values. The iterations were set to 70000, and the initial 30000 iterations, with a thinning factor of 10. Furthermore, the convergence of the model was diagnosed using a trace plot, density plot, and Brooks-Gelman-Rubin diagnosis plot (25). Finally, we calculated the RR and corresponding 95%CI, and the surface under the cumulative ranking (SUCRA) probabilities were used to rank the efficacy of various interventions (27). The value of the SUCRA is between 0 and 1 (0 ≤ SUCRA ≤ 1). When the SUCRA was 1, the intervention was effective, whereas when the SUCRA was 0, the intervention was ineffective. Subgroup analysis was performed according to EM severity (mild, moderate, and severe). Only 29 of the 31 RCTs included had myoclonus classification; therefore, a subgroup analysis was performed.

Song et al. pointed out that indirect comparison and NMA often involved three basic assumptions: homogeneity, similarity, and consistency hypothesis (28). In this study, we used the “node-splitting technique” to evaluate network consistency (29). A P value > 0.05 indicated consistency, and as such, we combined the direct and indirect estimates in the comparison of mixed treatment.

## Results

### Search Results

**FIGURE 1:**
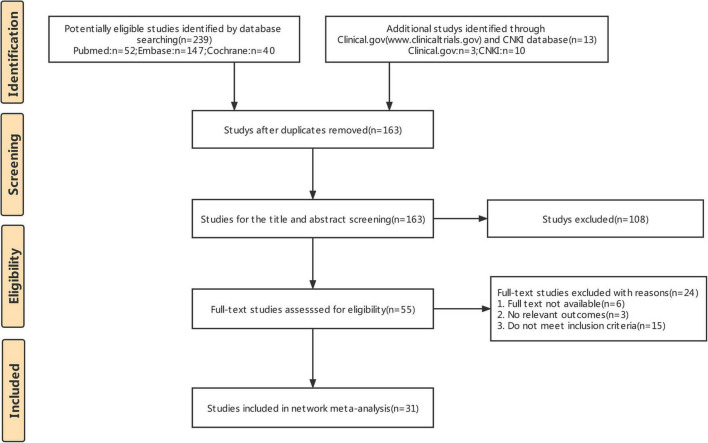
Flow diagram of the literature search and study.

The literature retrieval results and screening process are shown in Figure 1. A total of 251 studies were initially retrieved. Of these, 89 duplicate studies were removed using the EndNote software. A total of 108 studies were excluded after reading the titles and abstracts. Based on the full-text reviews, 24 studies were further excluded for various reasons: 15 did not meet the inclusion criteria or had incomplete information, and the full text of 6 records was not available. Finally, 31 RCTs (3209 patients) were included in this study.

### Characteristics of Included Studies

An overview of the selected studies is shown in Table 1. Most patients were scheduled for elective surgery under general anesthesia, with ASA physical status ranging from I to IV. During the induction of general anesthesia, the injection dosage of etomidate was 0.2–0.3 mg/kg, which is a commonly used induction dose in clinical practice. While there was a wide variety of drugs studied, for drugs with similar pharmacological effects, we categorized them as a group for analysis. Oxycodone, fentanyl, sufentanil, and remifentanil are all μ opioid agonists (μ-R agonists). Butorphanol, dezocine, and nalbuphine are κ opioid agonists predominate (κ-R agonists), and magnesium sulfate and ketamine are N-methyl-D-aspartic acid receptor antagonists (NMDA-R antagonists). The sample size of the 31 studies ranged from 45 to 284.

**TABLE 1:** Characteristics of included studies.

Author,year	Type of surgery	Age	ASA status	Induction dose of etomidate	Treatment	Case	No EM	Mild EM	Moderate- Severe EM
Wu, (55)	Elective surgery	18-65	I-II	0.3mg/kg	Placebo	52	13	8	31
					Ketamine 0.5mg/kg	52	40	7	5
Wang, (58)	Elective surgery	22-64	I-II	0.3mg/kg	Placebo	54	15	10	29
					Oxycodone 0.1mg/kg	54	54	0	0
					Fentanyl 1ug/kg	54	37	4	13
Sedighinejad, (42)	Orthopedic surgery	19-59	I-II	0.3mg/kg	Etomidate 0.03mg/kg	71	41	12	18
					Remifentanil 1ug/kg	71	30	12	29
					Midazolam 0.015mg/kg	71	20	3	48
					Magnesium sulfate 30mg/kg	71	10	4	57
Hwang, (51)	Elective surgery	Adults	I-II	0.3mg/kg	Placebo	30	7	6	17
					Remifentanil 1ug/kg	29	24	3	2
					Midazolam 0.5mg/kg	30	25	5	0
Mullick, (43)	Elective surgery	18-60	I-II	0.3mg/kg	Placebo	63	10	10	43
					Etomidate 0.03mg/kg	63	25	15	23
Mizrak, (59)	Day case surgery	18-60	I-II	0.3mg/kg	Placebo	30	11	7	12
					Dexmedetomidine 0.5ug/kg	30	20	5	5
Miao, (60)	Elective surgery	Adults	I-II	0.3mg/kg	Placebo	50	18	8	24
					Dexmedetomidine 0.5ug/kg	50	37	7	6
Alipour, (61)	Elective eye surgery	Adults	II-III	0.3mg/kg	Sufentanil 0.2ug/kg	25	18	2	5
					Midazolam 0.015mg/kg	25	4	0	21
An, (62)	Elective surgery	18-65	I-II	0.3mg/kg	Placebo	60	22	22	16
					Oxycodone 0.05mg/kg	60	45	10	5
Luan, (63)	Elective surgery	18-60	I-II	0.3mg/kg	Placebo	30	11	10	9
					Dexmedetomidine 0.5ug/kg	30	19	9	2
					Dexmedetomidine 1ug/kg	30	21	8	1‘
He^1^, (47)	Elective surgery	20-65	I-II	0.3mg/kg	Placebo	54	11	7	36
					Butorphanol	54	47	3	4
Guler, (64)	Elective surgery	Adults	I-III	0.2mg/kg	Placebo	25	7	6	12
					Ketamine 0.2mg/kg	25	9	10	6
					Ketamine 0.5mg/kg	25	7	10	8
					Magnesium sulfate 60mg	25	19	1	5
Gultop, (65)	Elective surgery	Adults	I-II	0.3mg/kg	Placebo	30	5	3	22
					Lidocaine 20mg	30	13	2	15
Gupta^1^, (46)	Laparoscopic cholecystectomy	20-60	I-II	0.3mg/kg	Placebo	50	14	6	30
					Nalbuphine,0.2mg/kg	50	40	6	4
Gupta^2^, (66)	Elective surgery	20-60	I-II	0.3mg/kg	Placebo	50	12	6	32
					Lidocaine 0.5mg/kg	50	20	5	25
					Lidocaine 1.0mg/kg	50	29	7	14
					Lidocaine 1.5mg/kg	50	23	9	18
He^2^, (48)	Elective surgery	20-65	I-II	0.3mg/kg	Placebo	54	13	7	34
					Dezocine 0.1mg/kg	54	54	0	0
Hüter, (67)	Elective cardioversion	Adults	III-IV	0.3mg/kg	Placebo	20	10	6	4
					Midazolam 0.015mg/kg	20	18	2	0
Aktolga, (68)	Not mentioned	Adults	I-III	A sleep dose of etomidate	Placebo	51	5	28	18
					Midazolam 0.5mg/kg	51	32	16	3
					Dexmedetomidine 1ug/kg	50	35	11	4
Ko, (69)	Elective surgery	65-74	I-II	0.2mg/kg	Placebo	30	16	8	6
					Fentanyl 1ug/kg	30	28	2	0
					Remifentanil 1ug/kg	30	29	1	0
Lv^1^, (49)	Elective surgery	Adults	I-II	0.3mg/kg	Placebo	40	14	3	23
					Dezocine 0.1mg/kg	40	28	5	7
Lv^2^, (57)	Elective hysteroscopy	20-55	I-II	0.3mg/kg	Placebo	43	5	13	25
					Sufentanil 0.1ug/kg	43	17	12	14
Woo, (70)	Plastic surgery	Adults	I	0.3mg/kg	Placebo	30	5	5	20
					Remifentanil 0.5ug/kg	30	27	3	0
					Remifentanil 1ug/kg	30	25	5	0
Prakash, (71)	Elective surgery	Adults	I-II	0.3mg/kg	Fentanyl 2ug/kg	70	36	11	23
					Midazolam 0.03mg/kg	70	15	10	45
Ilke, (72)	Various operations under general anesthesia	Adults	I-II	0.3mg/kg	Placebo	20	3	4	13
					Fentanyl 1ug/kg	20	12	2	6
					Midazolam 0.03mg/kg	20	6	1	13
Singh, (73)	Elective surgery	Adults	I-II	0.3mg/kg	Placebo	25	6	8	11
					Lidocaine 20mg	25	14	6	5
					Midazolam 1mg	25	18	4	3
Boztug, (74)	Not mentioned	Adults	I-II	0.3mg/kg	Placebo	15	3	1	11
					Remifentanil 0.5ug/kg	15	13	1	1
					Remifentanil 1ug/kg	15	14	1	0
Yukselen, (75)	Not mentioned	Adults	III	0.3mg/kg	Placebo	20	2	6	12
					Remifentanil 1ug/kg	20	19	1	0
					Fentanyl 1ug/kg	20	6	8	6
Yılmaz Çakirgöz, (33)	Elective surgery	18-60	I-II	0.3mg/kg	Placebo	25	6	7	12
					Gabapentin 400mg	25	11	8	6
					Gabapentin 800mg	25	18	2	5
					Gabapentin 1200mg	25	17	3	5
Choi, (53)	Elective surgery	Adults	I-III	0.3mg/kg	Placebo	54	20	18	16
					Rocuronium 0.06mg/kg	56	42	12	2
Un, *(56)	Elective surgery	Adults	I-II	0.3mg/kg	Placebo	50	22	TNM = 28	
					Magnesium sulfate 60mg	50	37	TNM = 13	
Mutlu, *(76)	Minor surgery or	Adults	I-II	0.3mg/kg	Placebo	30	26	TNM = 4	
	procedures				Remifentanil 1ug/kg	30	30	TNM = 0	
					Remifentanil 0.75ug/kg	30	30	TNM = 0	
					Remifentanil 0.5ug/kg	30	25	TNM = 5	

*ASA status = American Society of Anesthesiologists physical status; EM = Etomidate- induced myoclonus; TNM = Total number of myoclonus cases.*

*The presence of myoclonus was only reported as “present/absent”, and no gradation was performed.

### Pairwise Meta−Analysis and Network Meta-Analysis Results

**FIGURE 2:**
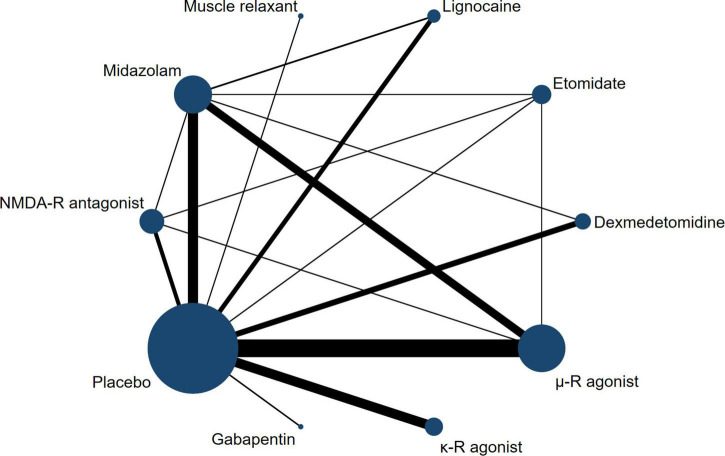
Network plot of treatment comparisons. The width of the line represents the number of RCTs per pairwise comparison, and the size of each node is proportional to the number of sample size.

The network relationship between different treatment regimens and placebo is shown in Figure 2. In our NMA, there were 26 two-arm studies, 4 three-arm studies, and 1 four-arm study, and a comparison between 10 interventions-, including placebo, was performed. μ-R agonists were most frequently included for comparisons, followed by midazolam, and NMDAR antagonists. Regarding heterogeneity, we compared the fixed-effects model with the random-effects model, and the results showed that the latter had smaller DIC and I^2^ values. Therefore, based on the heterogeneity analysis and DIC comparison, all data were analyzed using a consistent random-effects model. After 70000 iterations, the fluctuation of the four Markov chains was small, the trace plot and density plot tended to be stable, and the PSRF was close to 1, indicating satisfactory convergence of the model and relatively stable results (Supplementary Figure 1).

**FIGURE 3:**
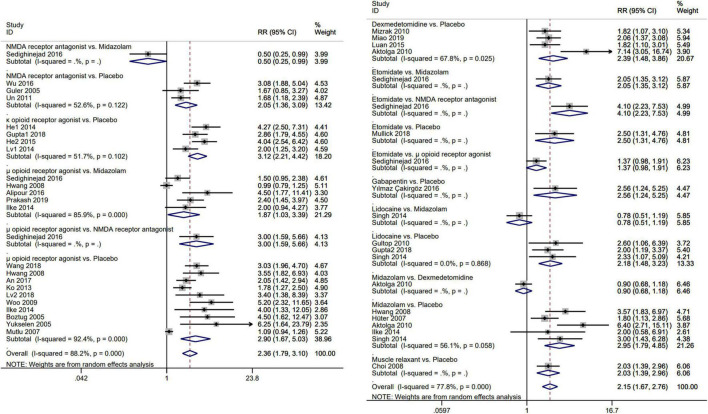
Forest plot for direct comparison of each pair of interventions. Meta-analysis use RR and 95%CI for the incidence of etomidate-induced myoclonus. *RR*, risk ratio; *CI*, confidence interval.

**FIGURE 4:**
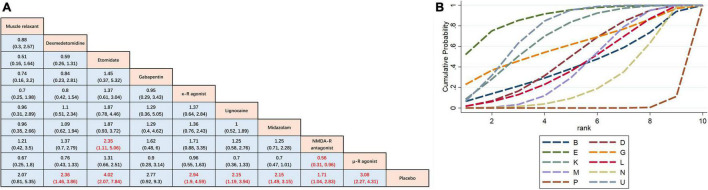
Network meta-analysis comparison. **(A)** The incidence of etomidate-induced myoclonus was analyzed by using RR and 95%CI. Data in each cell are RR (95%CI) for the comparison of column-defining treatment versus row-defining treatment. Significant results are highlighted in red. *RR*, risk ratio; *CI*, confidence interval. **(B)** Graphical ranking based on SUCRA values (Incidence of total EM). The numbers on the X-axis represent the rankings. As the numbers increases, the effectiveness of the interventions decreases. *B* muscle relaxant, *D* dexmedetomidine, *E* etomidate, *G* gabapentin, *P* placebo, *K* κ opioid receptor agonist, *L* lidocaine, *M* midazolam, *U* μ opioid receptor agonist, *N* NMDA receptor antagonist.

The results of the pairwise meta-analysis are shown in Figure 3. The results produced by NMA are illustrated in Figure 4A. In comparison with placebo, overall myoclonus incidence was significantly reduced after low-dose dexmedetomidine (RR 2.4, 95%CI 1.5–3.9), etomidate (RR 4.0, 95%CI 2.1–7.8), NMDA-R antagonist (RR 1.7, 95%CI 1.0–2.8), lidocaine (RR 2.1, 95%CI 1.2–3.9), midazolam (RR 2.2, 95%CI 1.5–3.2), μ-R agonist (RR 3.1, 95%CI 2.3–4.3), and κ-R agonist (RR 2.9, 95%CI 1.9–4.6) before induction of anesthesia. Gabapentin (RR 2.8, 95%CI 0.92–9.3) and muscle relaxants (RR 2.1, 95%CI 0.81–5.3) did not significantly reduce the overall incidence of EM. Additionally, etomidate (RR 2.35, 95%CI 1.11–5.06) was significantly better than the NMDAR antagonist, NMDAR antagonist (RR 0.56, 95%CI 0.31–0.96) was significantly worse than the μ-R agonist, and the differences among other drugs were not statistically significant. To further understand the results, the nine interventions were ranked by the SUCRA value. The higher the SUCRA value, the lower the incidence of myoclonus after etomidate induction. The corresponding SUCRA values are shown in Figure 4B. The results suggest that preoperative administration of low doses of opioids and etomidate is preferable to other regimens.

### Study Quality

Node-splitting technology was used to test the consistency of indirect and direct evidence, and the results are shown in Supplementary Figure 2. In the vast majority of comparisons, there was no statistically significant inconsistency between the direct and indirect estimates (*P* > 0.05). Publication bias was visually inspected using comparison-adjusted funnel plots (Supplementary Figure 3). Most studies were distributed on both sides of the midline, and the left and right distributions were roughly symmetrical, suggesting that there was little possibility of publication bias and a small sample effect.

The risk of bias for the 31 RCTs is presented in Supplementary Figure 4. A total of 21 studies described the generation of random sequences, 23 trials described concealment details, 29 studies blinded subjects, 28 trials blinded evaluators of outcomes, and all the included studies showed complete data. One study was judged to be high-risk because of the different baseline data (the induction dose of etomidate was statistically different between different groups). The GRADE assessment showed that the quality of evidence for etomidate compared to other interventions was “high,” indicating that the effect of using small doses of etomidate pre-induction to prevent EM is likely supported. The quality of evidence for the other comparisons is detailed in Supplementary Table 1.

### Subgroup Analysis of Myoclonus of Different Degrees

**FIGURE 5:**
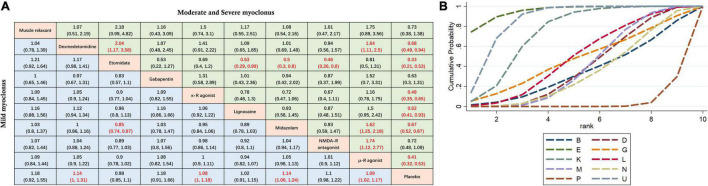
Subgroup analysis of myoclonus of different degrees. **(A)** The incidence of myoclonus of different severity after etomidate induction was analyzed by using RR and 95%CI. Data in each cell are RR (95%CI) for the comparison of column-defining treatment versus row-defining treatment. Significant results are highlighted in red. *RR*, risk ratio; *CI*, confidence interval. **(B)** Graphical ranking based on SUCRA values (Incidence of moderate to severe EM). The numbers on the X-axis represent the rankings. As the numbers increases, the effectiveness of the interventions decreases. *B* muscle relaxant, *D* dexmedetomidine, *E* etomidate, *G* gabapentin, *P* placebo, *K* κ opioid receptor agonist, *L* lidocaine, *M* midazolam, *U* μ opioid receptor agonist, *N* NMDA receptor antagonist.

Mild myoclonus is a brief movement of a part of the body, such as the fingers and wrist. Moderate myoclonus is usually a movement of two different muscles, such as the face, leg, shoulder or elbow, with pronounced tremors. Severe myoclonus is the intense movement or rigidity of two muscles; for example, the body undergoes fast abduction (30). Subgroup analysis was conducted based on the severity of myoclonus. Here, we mainly divided the patients into two groups: mild myoclonus and moderate-to-severe myoclonus. The results showed that preoperative low doses of etomidate (RR 0.33, 95%CI, 0.21–0.53), μ-R agonist (RR 0.41, 95%CI 0.32–0.53), κ-R agonist (RR 0.48, 95%CI 0.35–0.65), dexmedetomidine (RR 0.68, 95%CI 0.49–0.94), midazolam (RR 0.67, 95%CI 0.52–0.87), and lidocaine (RR 0.62, 95%CI 0.41–0.93) significantly reduced the incidence of moderate to severe myoclonus compared with placebo, but only dexmedetomidine (RR 1.14, 95%CI 1–1.31), midazolam (RR 1.14, 95%CI 1.06–1.24), κ-R agonist (RR 1.08, 95%CI 1–1.18), and μ-R agonist (RR 1.09, 95%CI 1.02–1.17) reduced the incidence of mild myoclonus. The results of the subgroup analysis are shown in Figure 5A. Since moderate-to-severe myoclonus is the most common clinical problem, we focused on the prevention and treatment effects of various interventions on moderate-to-severe myoclonus. For effectiveness in preventing moderate to severe EM, Figure 5B shows the corresponding ranking based on SUCRA values: etomidate > μ-R agonist > κ-R agonist > lidocaine > gabapentin > midazolam > dexmedeto midine > muscle relaxant > NMDA-R antagonist.

## Discussion

Our NMA attempted to summarize the available data using direct and indirect evidence to conclude that pre-induction of anesthesia with low-dose opioids and etomidate is the best intervention to reduce the incidence and severity of etomidate-induced myoclonus. However, further research is warranted.

As a fast-acting intravenous anesthetic, etomidate has a low risk of hemodynamic damage. Compared with propofol, etomidate is especially suitable for anesthesia, procedural sedation and analgesia (PSA) in the emergency department for patients with trauma, shock, and acute abdomen, with hemodynamic instability. However, in some areas, etomidate use is partially limited by its ability to cause adrenocortical depression, myoclonus, and injection pain (31). Etomidate can induce myoclonus which may increase the risk of aspiration in satiated patients and patients with a decreased cardiac reserve and increased cardiac oxygen consumption (32). During severe myoclonus, electrocardiogram electrode shifts and pulse oxygen saturation measurements often show desaturation (33). Myoclonus has been reported to be associated with hypoxemia during spontaneous ventilation when etomidate was used in the emergency department for PSA (34). In summary, myoclonic events may be large enough to delay patient monitoring and evaluation of intervention success.

The anesthetic effects of etomidate and its derivatives are generally thought to occur *via* GABA_*A*_ receptors (35). Our current understanding of the mechanisms of etomidate-induced myoclonus is fragmented, contradictory, and confusing. Modica and Gancher et al. noted that etomidate is an electroencephalogram drug that has been shown to cause epileptic activities in non-epileptic patients (36–38). Therefore, etomidate-induced myoclonus may occur as epileptic activities, similar to the mechanisms underlying epilepsy. In contrast, Doenicke et al., in their study, reported that after giving etomidate, part of myoclonus patients only can be observed in EEG amplitude smaller, isolated, rapid, sharp transient wave, different from the typical epileptic EEG activity (30). Epilepsy is a clinical event with a definitive EEG diagnosis accompanied by a widespread, diffuse wave of EEG activities (39). It is prudent to say that anesthetics usually induce epileptiform activity, but rarely seizures. Epileptiform activity differs from epilepsy in that it primarily refers to the hypersynchrony of neurons in a small area (< 1 cm^2^), and is considered an indicator of an incipiently unstable neocortex, with a weak association with clinically meaningful seizures (40). Another theory is that etomidate-induced myoclonus is a disinhibitory phenomenon (41). It may be that there are differences in local cerebral blood flow or in the affinity of receptors in the central nervous system that cause the action of etomidate to become unsynchronized. For example, large quantities of etomidate tend to inhibit cortical activity before they inhibit subcortical activity (30). Subsequently, subcortical disinhibition makes the pathways associated with controlling skeletal muscles more sensitive to spontaneous neurotransmitters, causing spontaneous myoclonus. GABAergic synaptic excitation and subgroup specificity between interneurons, which control the output of pyramidal cells, also partly explain this remarkable neurophysiological phenomenon (40). Although myoclonic excitation is not thought to be caused by epileptic foci, drugs such as dexmedetomidine (α-2 agonist-mediated reduction of convulsion severity) and gabapentin (antiepileptic agents that increase the inhibitory effect of GABA) effectively reduce EM (33).

Although knowledge gaps still remain, it seems that implementing effective prevention is crucial and of the most practical value. As a single large dose of etomidate inhibits cortical activity earlier than subcortical activity, myoclonus can be prevented by prior suppression of subcortical neuronal activity with known drugs. Among the results of our analysis, seven interventions (μ-R agonist, κ-R agonist, etomidate, dexmedetomidine, midazolam, NMDA-R antagonist, and lidocaine) showed statistically significant improvements in preventing the incidence of EM compared with placebo, and six interventions (μ-R agonist, κ-R agonist, etomidate, dexmedetomidine, midazolam, and lidocaine) showed statistically significant improvements in preventing the incidence of moderate to severe EM.

The distinct distribution of GABA_*A*_ receptor subunits (mainly β subunits) explains the dose-dependent effects of etomidate on the central nervous system. Etomidate can inhibit subcortical inhibitory circuits earlier and at lower doses, and when large doses are administered simultaneously, this mismatch is exaggerated, producing clinically visible myoclonus (32, 42, 43). Therefore, small doses of etomidate pre-induction can reduce the incidence of myoclonus.

The role of the κ-opioid receptor as a neuronal excitatory modulator is well known. Activation of the κ receptor reduces glutamate release, produces postsynaptic hyperpolarization, and inhibits seizure activity (44). κ-opioid receptor agonists also interact with a variety of neurotransmitter systems (μ opioid receptor, δ opioid receptor, γ-aminobutyric acid-benzodiazepine-chloride ion channel, GABA receptors, and NMDA receptor). Dezocine, butorphanol, and nalbuphine mainly bind to and regulate κ-opioid receptors; therefore, the mechanism by which these drugs reduce etomidate-induced myoclonus may lie in their activation through κ receptor regulation as agonists (45–49).

Benzodiazepines and opioids, such as fentanyl, are known to inhibit subcortical neuronal activity (38). Many randomized controlled trials have shown that multiple opioids, including fentanyl, sufentanil, remifentanil, and oxycodone, are effective in reducing the incidence and severity of EM. However, apnea, nausea, vomiting, and bradycardia are possible (46, 50, 51). In the study by Su et al., intramuscular injection of midazolam (0.05 mg/kg) 30 min before etomidate injection did not reduce the incidence of myoclonus, which was not significantly different from the previously reported incidence of myoclonus (50). Since opioid receptors are widely distributed in the brain, the mechanism by which opioid agonists inhibit myoclonus remains unknown. It may be that μ-opioid receptors are stimulated in the basal ganglia, which changes the function of GABA receptors and reduces the release of GABA, thus inhibiting subcortical neuronal activity. In Parkinson’s-related studies, opioid neuropeptides have been reported to strongly regulate synaptic transmission and striatal projection neuron (SPNs) activity (52). High opioid levels occur in parallel with abnormal dopaminergic transmission, producing symptoms similar to increased dopamine levels, thus attenuating the onset of muscle fibrillation.

Although other studies were included in a supplementary analysis, the reduction in myoclonic symptoms was not as significant as opioid and etomidate preconditioning, as indicated by our results. Non-depolarizing muscle relaxants are associated with blocking nerve conduction at neuromuscular junctions (53). Lidocaine reduces the activity of the nerve centers that cause myoclonus (54). Magnesium sulfate and ketamine are non-competitive N-methyl-D-aspartate receptor antagonists, and their myoclonic inhibition is thought to be related to the inhibition of NMDA receptor activity in the central nervous system (42, 55). However, the efficacy varies from study to study. In the study by Un et al., the incidence of EM after magnesium sulfate pretreatment was 26%, whereas in the study by Sedighinejad et al., the incidence was as high as 86% (42, 56).

Regarding the response rates before and after the entire NMA, the hierarchical order of total myoclonus incidence differed slightly from the results of the subgroup analysis, but opioid or etomidate preconditioning still showed a significant advantage. The latter seems to be the more important result.

There were some limitations to our study. First, fewer than 60% of the studies included more than 100 participants, which could have contributed to the risk of bias. Second, SUCRA was used to estimate the rank probability of comparative efficacy between different interventions. However, it has limitations, all of which are subject to uncertainties, and thus, the results need to be interpreted with caution. Third, in the past, most studies had different drug dosages according to the different curative effects. However, in this study, we only limited the category of drugs. We did not limit the dosage. This may have caused a bias. Fourth, this study did not consider the safety of using these drugs because only a few studies reported adverse reactions and there was not a large amount of data available. Fifth, although transcutaneous acupoint electrical stimulation has been previously reported to reduce the incidence and severity of etomidate myoclonus (57), non-pharmacological or other interventions were not considered in our study. Sixth, 31 RCTs were included, including nine interventions. However, only seven were studied in two or more trials. Only two studies reported on both muscle relaxants and gabapentin, respectively, which explains the wide 95% confidence interval for the ultimate RR for both drugs.

## Conclusion

Based on the currently available evidence, we used the NMA approach to compare the impact of different interventions on EM for the first time. Taken together, preoperative low-dose etomidate is the best intervention for preventing severe general myoclonus. Although opioids also have a prophylactic effect on general myoclonus, side effects should not be ignored. Our study provides strong evidence that implicates clinical practice. In particular, Etomidate may have an even more important role in clinical intravenous anesthesia.

## Data Availability Statement

The original contributions presented in the study are included in the article/Supplementary Material, further inquiries can be directed to the corresponding author/s.

## Author Contributions

K-DZ proposed the research idea, developed a retrieval strategy, and drafted the manuscript. L-YW and K-DZ conducted a literature search, literature selection, and bias risk assessment. D-XZ and Z-HZ performed data extraction. H-LW reviewed and revised the manuscript. All authors contributed to the analysis and interpretation of the data, revised the manuscript, and approved the final version prior to submission.

## Conflict of Interest

The authors declare that the research was conducted in the absence of any commercial or financial relationships that could be construed as a potential conflict of interest.

## Publisher’s Note

All claims expressed in this article are solely those of the authors and do not necessarily represent those of their affiliated organizations, or those of the publisher, the editors and the reviewers. Any product that may be evaluated in this article, or claim that may be made by its manufacturer, is not guaranteed or endorsed by the publisher.

## Footnotes

1
http://mcmc-jags.sourceforge.net/
