# Supplementary material for: Comparison of the Effectiveness of Various Drug Interventions to Prevent Etomidate-Induced Myoclonus: A Bayesian Network Meta-Analysis
Source: Front Med (Lausanne). 2022 Apr 26;9:799156. doi: 10.3389/fmed.2022.799156 (PMC9086535; doi:10.3389/fmed.2022.799156)
Supplement: Supplementary file 1 [file Data_Sheet_1.ZIP › Supplementary Material Presentation/Supplementary_Material.docx]

Supplementary Material

# Supplementary Data

# 1.1 Supplementary Appendix 1. The detailed search strategy

| **Database** | **Retrieval date** | **detailed search strategy** |
| --- | --- | --- |
| Pubmed | August 22, 2021 | **#1** ("Etomidate"[Mesh]) AND "Myoclonus"[Mesh]  **#2** ("Randomized Controlled Trial" [Publication Type] OR "Controlled Clinical Trial" [Publication Type] OR "Clinical Trials as Topic"[Mesh:NoExp] OR randomized[Title/Abstract] OR placebo  **#3** #1 AND #2 |
| Embase | August 22, 2021 | **#1** 'etomidate'/exp  **#2** 'myoclonus'/exp  **#3** #1 AND #2  **#4** 'crossover procedure'/exp OR 'double blind procedure'/exp OR 'randomized controlled trial'/exp OR 'single blind procedure'/exp  **#5** random*:ti,ab,kw OR factorial*:ti,ab,kw OR crossover*:ti,ab,kw OR ((cross NEXT/1 over*):ti,ab,kw) OR placebo*:ti,ab,kw OR ((doubl* NEAR/1 blind*):ti,ab,kw) OR ((singl* NEAR/1 blind*):ti,ab,kw) OR assign*:ti,ab,kw OR allocat*:ti,ab,kw OR volunteer*:ti,ab,kw  **#6** #4 OR #5  **#7** #3 AND #6 |
| Cochrane Central Register of Controlled Trials | August 22, 2021 | **#1** MeSH descriptor: [Etomidate] explode all trees  **#2** MeSH descriptor: [Myoclonus] explode all trees  **#3** #1 AND #2 |
| NIH ClinicalTrials.gov | August 22, 2021 | **Condition or disease:** Myoclonus  **Other terms:**  Etomidate  **Study type:**Interventional Studies(Clinical Trials) |
| CNKI (China National Knowledge Infrastructure) | August 22, 2021 | ( (主题=中英文扩展(Etomidate) 或者 题名=中英文扩展(Etomidate) 或者 v_subject=Etomidate 或者 title=Etomidate) 并且 (主题=中英文扩展(Myoclonus) 或者 题名=中英文扩展(Myoclonus) 或者 v_subject=Myoclonus 或者 title=Myoclonus) ) (模糊匹配) |

# 1.2 Supplementary Appendix 2. The details of 31 articles included in this NMA.

(1-31)

1. Aktolga S, Gunes Y, Gunduz M, Isik G. A comparison of midazolam and dexmedetomidine for the prevention of myoclonic movements and pain following etomidate injection %J Journal of Anaesthesiology Clinical Pharmacology. (2010) 26(2).

2. Alipour M, Tabari M, Azad AM. Comparative study evaluating efficacy of sufentanil versus midazolam in preventing myoclonic movements following etomidate. *Journal of anaesthesiology, clinical pharmacology* (2016) 32(1):29-32. Epub 2016/03/24. doi: 10.4103/0970-9185.173382. PubMed PMID: 27006537; PubMed Central PMCID: PMCPMC4784209.

3. An X, Li C, Sahebally Z, Wen X, Zhao B, Fang X. Pretreatment with Oxycodone Simultaneously Reduces Etomidate-Induced Myoclonus and Rocuronium-Induced Withdrawal Movements During Rapid-Sequence Induction. *Medical science monitor : international medical journal of experimental and clinical research* (2017) 23:4989-94. Epub 2017/10/20. doi: 10.12659/msm.902652. PubMed PMID: 29046518; PubMed Central PMCID: PMCPMC5659139.

4. Boztug N, Bigat Z, Onder G, Dikici A, Karsli B, Ertok E. The comparison of different doses of remifentanil on preventing myoclonus due to etomidate: A-504 %J European Journal of Anaesthesiology. (2005) 22 Suppl 34.

5. Choi JM, Choi IC, Jeong YB, Kim TH, Hahm KD. Pretreatment of rocuronium reduces the frequency and severity of etomidate-induced myoclonus. *Journal of clinical anesthesia* (2008) 20(8):601-4. Epub 2008/12/23. doi: 10.1016/j.jclinane.2008.06.010. PubMed PMID: 19100933.

6. Guler A, Satilmis T, Akinci SB, Celebioglu B, Kanbak M. Magnesium sulfate pretreatment reduces myoclonus after etomidate. *Anesthesia and analgesia* (2005) 101(3):705-9. Epub 2005/08/24. doi: 10.1213/01.Ane.0000160529.95019.E6. PubMed PMID: 16115978.

7. Gultop F, Akkaya T, Bedirli N, Gumus H. Lidocaine pretreatment reduces the frequency and severity of myoclonus induced by etomidate. *Journal of anesthesia* (2010) 24(2):300-2. Epub 2010/01/29. doi: 10.1007/s00540-010-0869-6. PubMed PMID: 20108006.

8. Gupta M, Gupta P. Nalbuphine pretreatment for prevention of etomidate induced myoclonus: A prospective, randomized and double-blind study. *Journal of anaesthesiology, clinical pharmacology* (2018) 34(2):200-4. Epub 2018/08/15. doi: 10.4103/joacp.JOACP_210_16. PubMed PMID: 30104829; PubMed Central PMCID: PMCPMC6066906.

9. Gupta P, Gupta M. Comparison of different doses of intravenous lignocaine on etomidate-induced myoclonus: A prospective randomised and placebo-controlled study. *Indian journal of anaesthesia* (2018) 62(2):121-6. Epub 2018/03/02. doi: 10.4103/ija.IJA_563_17. PubMed PMID: 29491517; PubMed Central PMCID: PMCPMC5827478.

10. He L, Ding Y, Chen H, Qian Y, Li Z. Butorphanol pre-treatment prevents myoclonus induced by etomidate: a randomised, double-blind, controlled clinical trial. *Swiss medical weekly* (2014) 144:w14042. Epub 2014/10/16. doi: 10.4414/smw.2014.14042. PubMed PMID: 25317545.

11. He L, Ding Y, Chen H, Qian Y, Li Z. Dezocine pretreatment prevents myoclonus induced by etomidate: a randomized, double-blinded controlled trial. *Journal of anesthesia* (2015) 29(1):143-5. Epub 2014/05/27. doi: 10.1007/s00540-014-1854-2. PubMed PMID: 24858231.

12. Hüter L, Schreiber T, Gugel M, Schwarzkopf K. Low-dose intravenous midazolam reduces etomidate-induced myoclonus: a prospective, randomized study in patients undergoing elective cardioversion. *Anesthesia and analgesia* (2007) 105(5):1298-302, table of contents. Epub 2007/10/26. doi: 10.1213/01.ane.0000287248.25610.c0. PubMed PMID: 17959958.

13. Hwang JY, Kim JH, Oh AY, Do SH, Jeon YT, Han SH. A comparison of midazolam with remifentanil for the prevention of myoclonic movements following etomidate injection. *The Journal of international medical research* (2008) 36(1):17-22. Epub 2008/01/31. doi: 10.1177/147323000803600103. PubMed PMID: 18230263.

14. Ilke I, Sinan U, Mehmet T, Ayşe V, Gökhan GY, Yilmaz IF, et al. Prevention of etomidate-induced myoclonus: which is superior: Fentanyl, midazolam, or a combination? A Retrospective comparative study. %J Medical science monitor : international medical journal of experimental and clinical research. (2014) 20.

15. Ko BJ, Oh JN, Lee JH, Choi SR, Lee SC, Chung CJ. Comparison of effects of fentanyl and remifentanil on hemodynamic response to endotracheal intubation and myoclonus in elderly patients with etomidate induction. *Korean journal of anesthesiology* (2013) 64(1):12-8. Epub 2013/02/02. doi: 10.4097/kjae.2013.64.1.12. PubMed PMID: 23372880; PubMed Central PMCID: PMCPMC3558642.

16. Luan HF, Zhao ZB, Feng JY, Cui JZ, Zhang XB, Zhu P, et al. Prevention of etomidate-induced myoclonus during anesthetic induction by pretreatment with dexmedetomidine. *Brazilian journal of medical and biological research = Revista brasileira de pesquisas medicas e biologicas* (2015) 48(2):186-90. Epub 2014/10/30. doi: 10.1590/1414-431x20144100. PubMed PMID: 25351237; PubMed Central PMCID: PMCPMC4321226.

17. Lv Y, He H, Xie J, Jin W, Shou C, Pan Y, et al. Effects of transcutaneous acupoint electrical stimulation combined with low-dose sufentanil pretreatment on the incidence and severity of etomidate-induced myoclonus: A randomized controlled trial. *Medicine* (2018) 97(23):e10969. Epub 2018/06/08. doi: 10.1097/md.0000000000010969. PubMed PMID: 29879048; PubMed Central PMCID: PMCPMC5999512.

18. Lv Z, Fang J, Zhu J, Liang B, Li F, Jiang S, et al. Intravenous dezocine pretreatment reduces the incidence and intensity of myoclonus induced by etomidate. *Journal of anesthesia* (2014) 28(6):944-7. Epub 2014/05/16. doi: 10.1007/s00540-014-1842-6. PubMed PMID: 24828848.

19. Miao S, Zou L, Wang G, Wang X, Liu S, Shi M. Effect of dexmedetomidine on etomidate-induced myoclonus: a randomized, double-blind controlled trial. *Drug design, development and therapy* (2019) 13:1803-8. Epub 2019/06/27. doi: 10.2147/dddt.S194456. PubMed PMID: 31239638; PubMed Central PMCID: PMCPMC6554000.

20. Mizrak A, Koruk S, Bilgi M, Kocamer B, Erkutlu I, Ganidagli S, et al. Pretreatment with dexmedetomidine or thiopental decreases myoclonus after etomidate: a randomized, double-blind controlled trial. *The Journal of surgical research* (2010) 159(1):e11-6. Epub 2009/12/19. doi: 10.1016/j.jss.2009.07.031. PubMed PMID: 20018300.

21. Mullick P, Talwar V, Aggarwal S, Prakash S, Pawar M. Comparison of priming versus slow injection for reducing etomidate-induced myoclonus: a randomized controlled study. *Korean journal of anesthesiology* (2018) 71(4):305-10. Epub 2018/08/04. doi: 10.4097/kja.d.18.27168. PubMed PMID: 30071713; PubMed Central PMCID: PMCPMC6078874.

22. Mutlu NM, Ediz N, Karabulut E, Gogus N. The effects of different doses of remifentanil pretreatment on etomidate injection pain and myoclonus: 9AP3-9 %J European Journal of Anaesthesiology. (2007) 24 Suppl 39.

23. Prakash S, Mullick P, Virmani P, Talwar V, Singh R. Effect of Pre-Treatment with a Combination of Fentanyl and Midazolam for Prevention of Etomidate-Induced Myoclonus %J Turk J Anaesthesiol Reanim. (2019) 49(1).

24. Sedighinejad A, Naderi Nabi B, Haghighi M, Biazar G, Imantalab V, Rimaz S, et al. Comparison of the Effects of Low-Dose Midazolam, Magnesium Sulfate, Remifentanil and Low-Dose Etomidate on Prevention of Etomidate-Induced Myoclonus in Orthopedic Surgeries. *Anesthesiology and pain medicine* (2016) 6(2):e35333. Epub 2016/06/02. doi: 10.5812/aapm.35333. PubMed PMID: 27247915; PubMed Central PMCID: PMCPMC4885461.

25. Singh K, Ruchi G, Singh A, Kaur B. Efficacy of lignocaine versus midazolam in controlling etomidate-induced myoclonus: a randomized placebo-controlled study %J Ain-Shams Journal of Anaesthesiology. (2014) 7(3).

26. Un B, Ceyhan D, Yelken B. Prevention of etomidate-related myoclonus in anesthetic induction by pretreatment with magnesium. *Journal of research in medical sciences : the official journal of Isfahan University of Medical Sciences* (2011) 16(11):1490-4. Epub 2012/09/14. PubMed PMID: 22973352; PubMed Central PMCID: PMCPMC3430068.

27. Wang W, Lv J, Wang Q, Yang L, Yu W. Oxycodone for prevention of etomidate-induced myoclonus: a randomized double-blind controlled trial. *The Journal of international medical research* (2018) 46(5):1839-45. Epub 2018/03/15. doi: 10.1177/0300060518761788. PubMed PMID: 29536782; PubMed Central PMCID: PMCPMC5991229.

28. Woo LS, Jue GH, Chul PS, Young KJ, Hyung KJ, Yeon LJ, et al. The effect of remifentanil for reducing myoclonus during induction of anesthesia with etomidate. %J Korean journal of anesthesiology. (2009) 57(4).

29. Wu GN, Xu HJ, Liu FF, Wu X, Zhou H. Low-Dose Ketamine Pretreatment Reduces the Incidence and Severity of Myoclonus Induced by Etomidate: A Randomized, Double-Blinded, Controlled Clinical Trial. *Medicine* (2016) 95(6):e2701. Epub 2016/02/13. doi: 10.1097/md.0000000000002701. PubMed PMID: 26871805; PubMed Central PMCID: PMCPMC4753901.

30. Yılmaz Çakirgöz M, Demirel İ, Duran E, Özer AB, Hancı V, Türkmen Ü A, et al. Effect of gabapentin pretreatment on myoclonus after etomidate: a randomized, double-blind, placebo-controlled study. *Brazilian journal of anesthesiology (Elsevier)* (2016) 66(4):356-62. Epub 2016/06/28. doi: 10.1016/j.bjane.2014.11.014. PubMed PMID: 27343784.

31. Yukselen AM, Guler A, Celebioglu B, Kanbak M, Aypar U. Comparison of the effect of remifentanil and fentanyl on myoclonus due to etomidate: A-507 %J European Journal of Anaesthesiology. (2005) 22 Suppl 34.

# Supplementary Figures and Tables


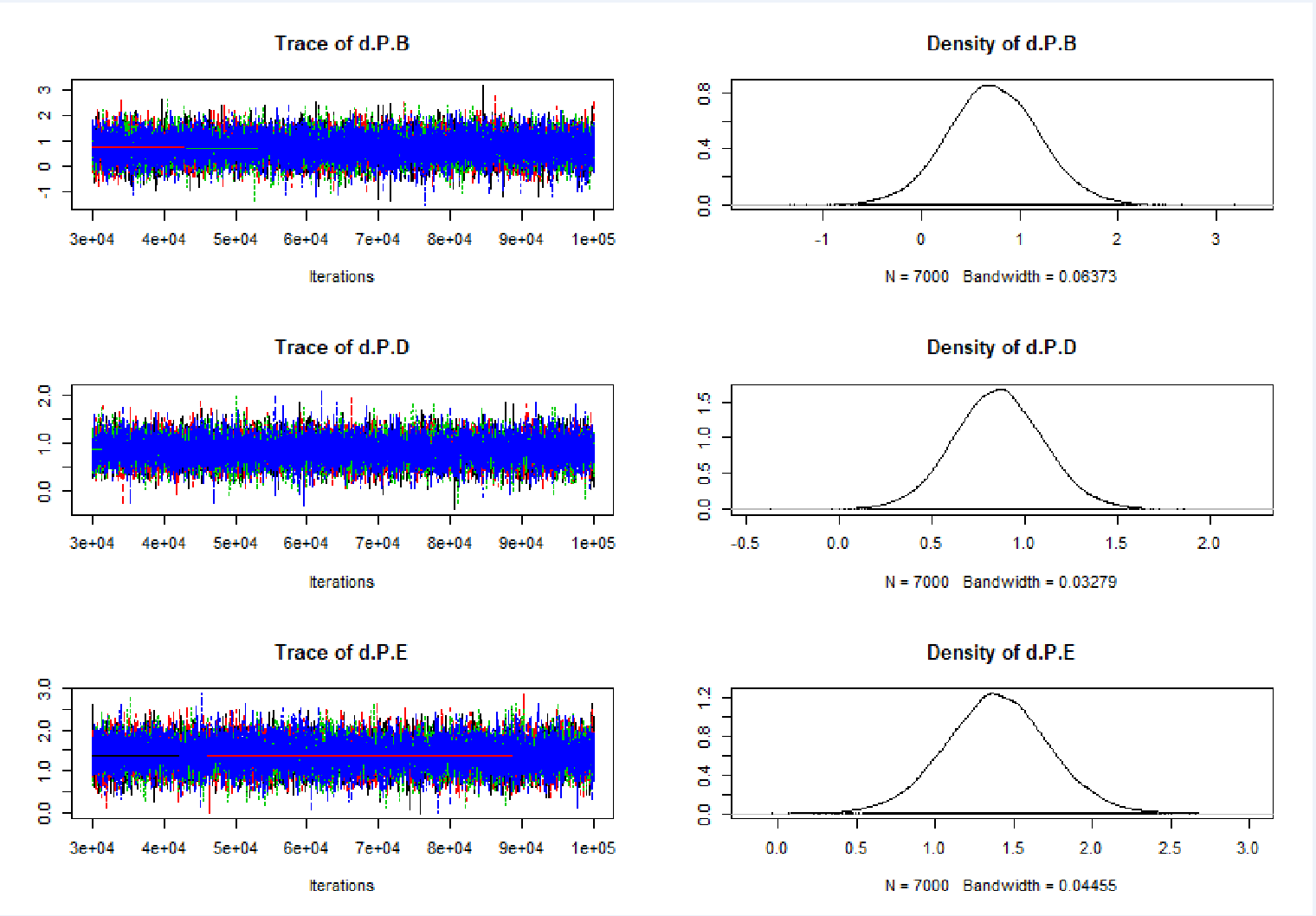


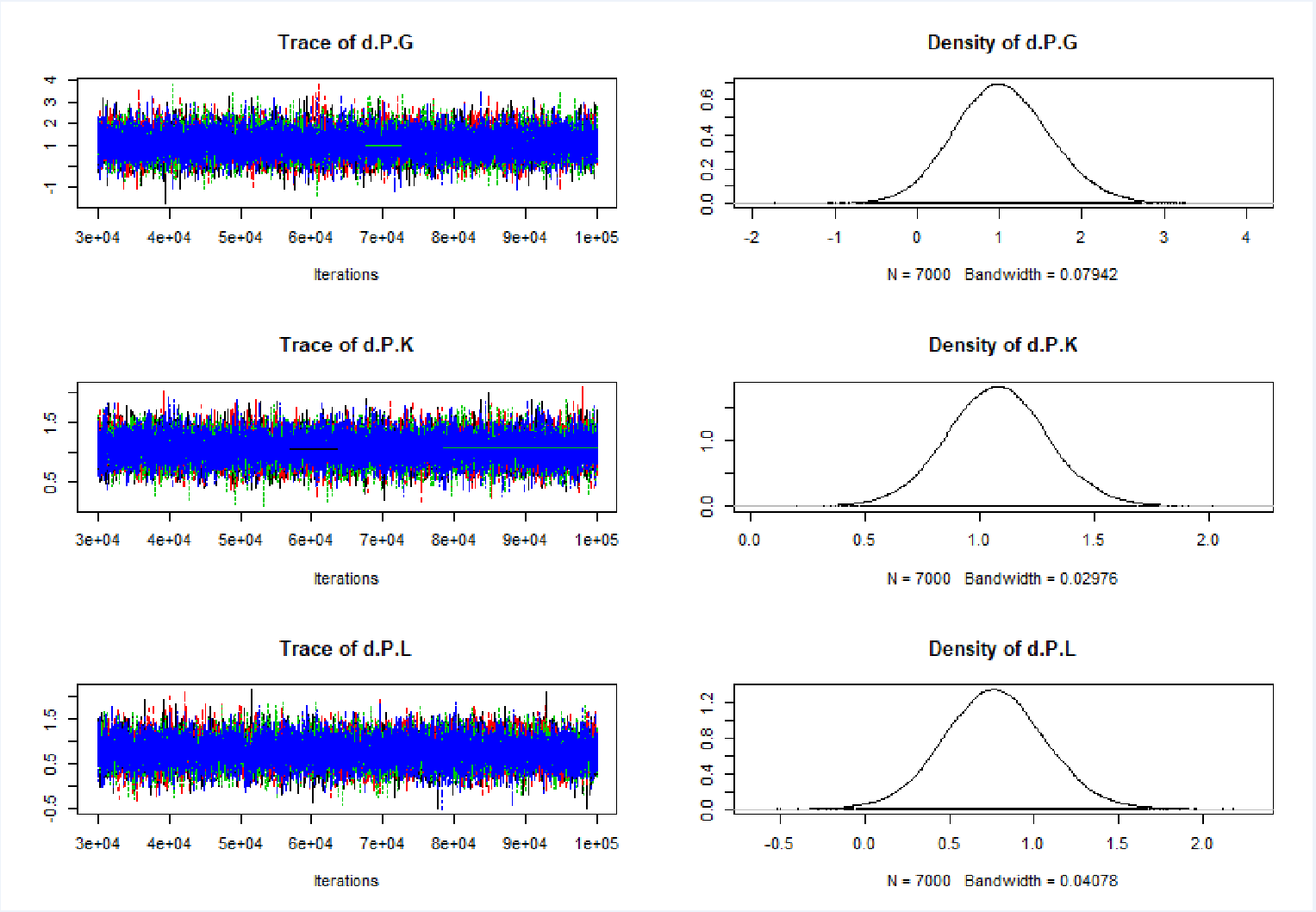


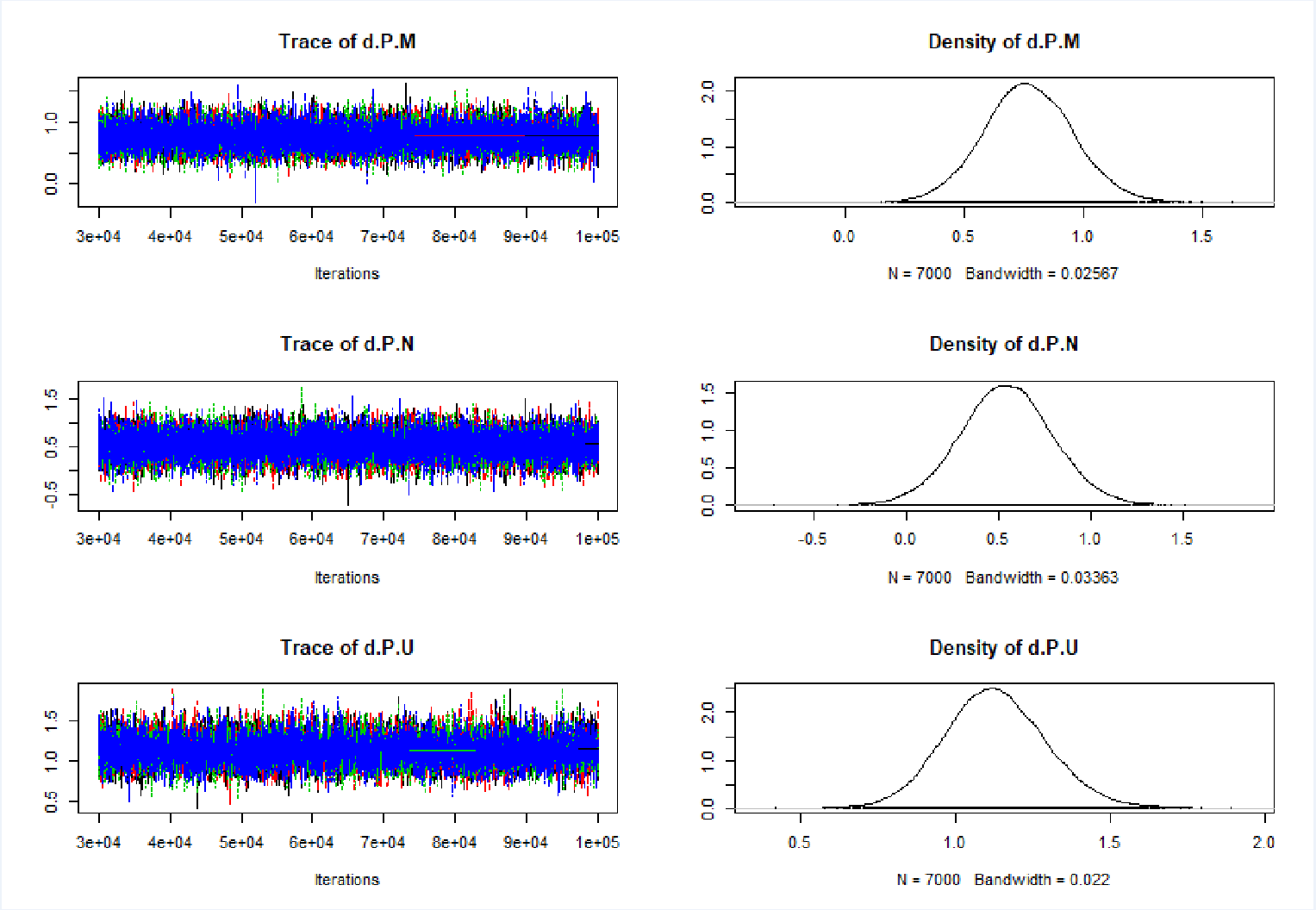


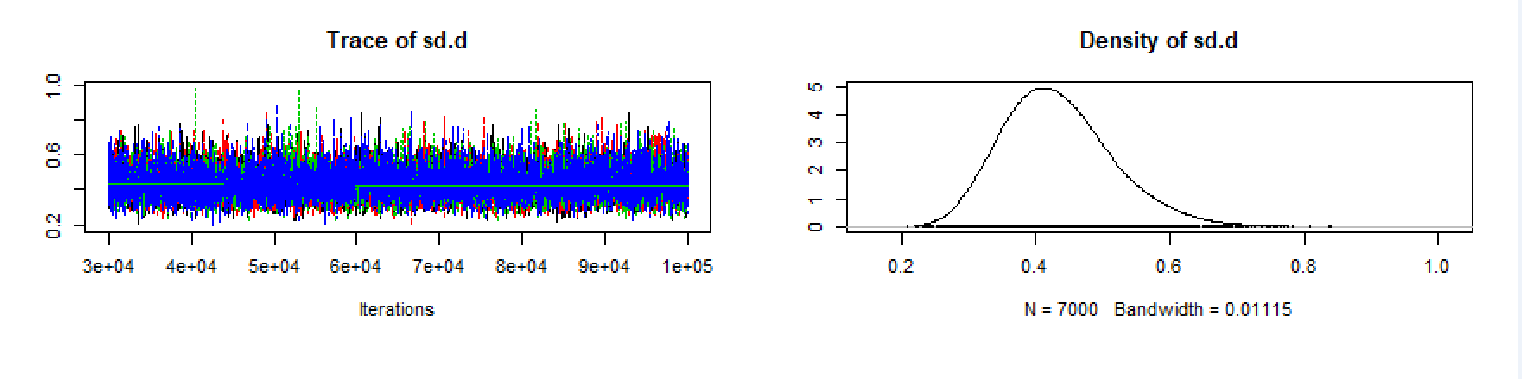


**Supplementary Figure 1.** Trace plot and density plot. The trace plot and density plot are used to diagnose the convergence degree of the model and judge whether the convergence degree is satisfactory. After 70000 iterations, the fluctuation of the four Markov chains is small, the trace plot and density plot tend to be stable.

(B) Muscle relaxant (D) Dexmedetomidine (E) etomidate (G) Gabapentin (P) Placebo (K) κ opioid receptor agonist (L) Lidocaine (M) Midazolam (U) μ opioid receptor agonist (N) NMDA receptor antagonist


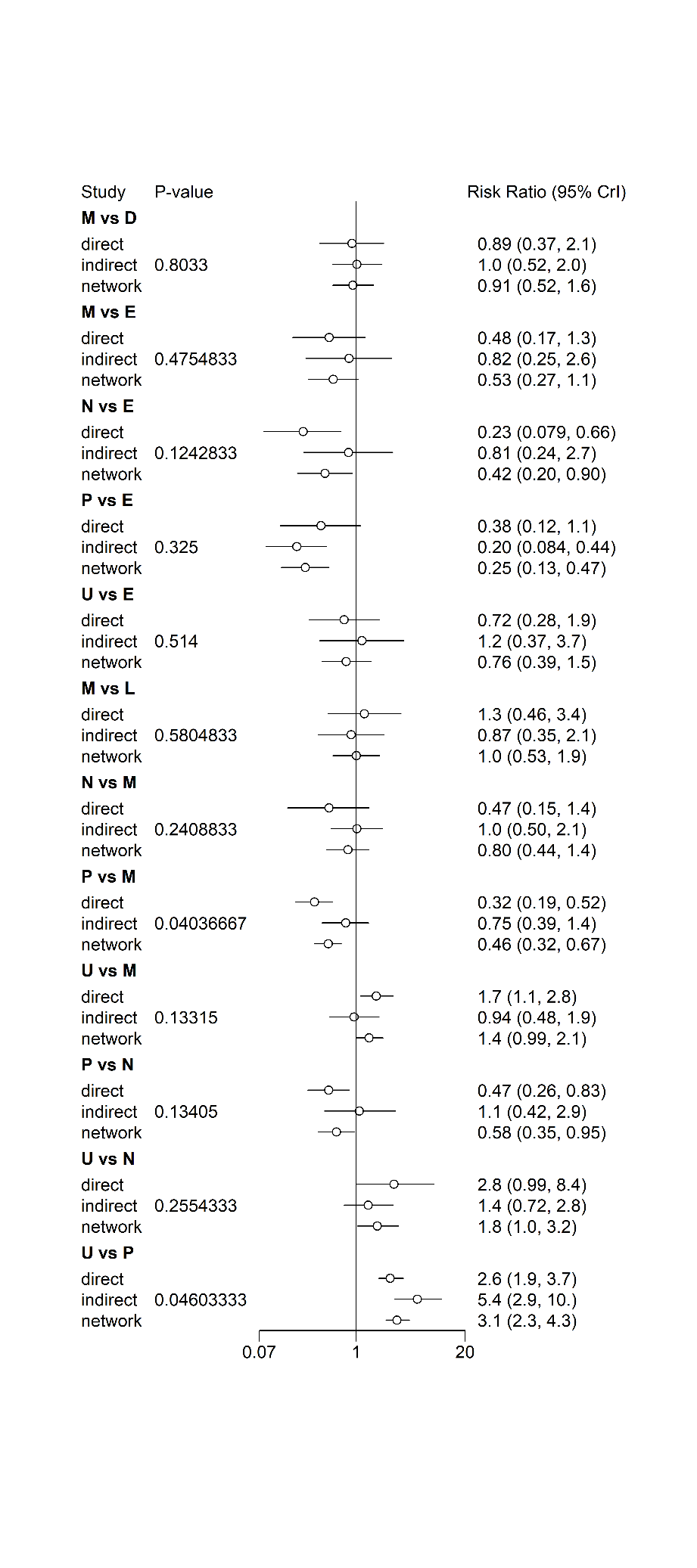


**Supplementary Figure 2.** Node-splitting results for each comparison. There appeared to be no statistically significant inconsistency between the direct and indirect estimates in all comparisons (P value >0.05).

(B) Muscle relaxant (D) Dexmedetomidine (E) etomidate (G) Gabapentin (P) Placebo (K) κ opioid receptor agonist (L) Lidocaine (M) Midazolam (U) μ opioid receptor agonist (N) NMDA receptor antagonist


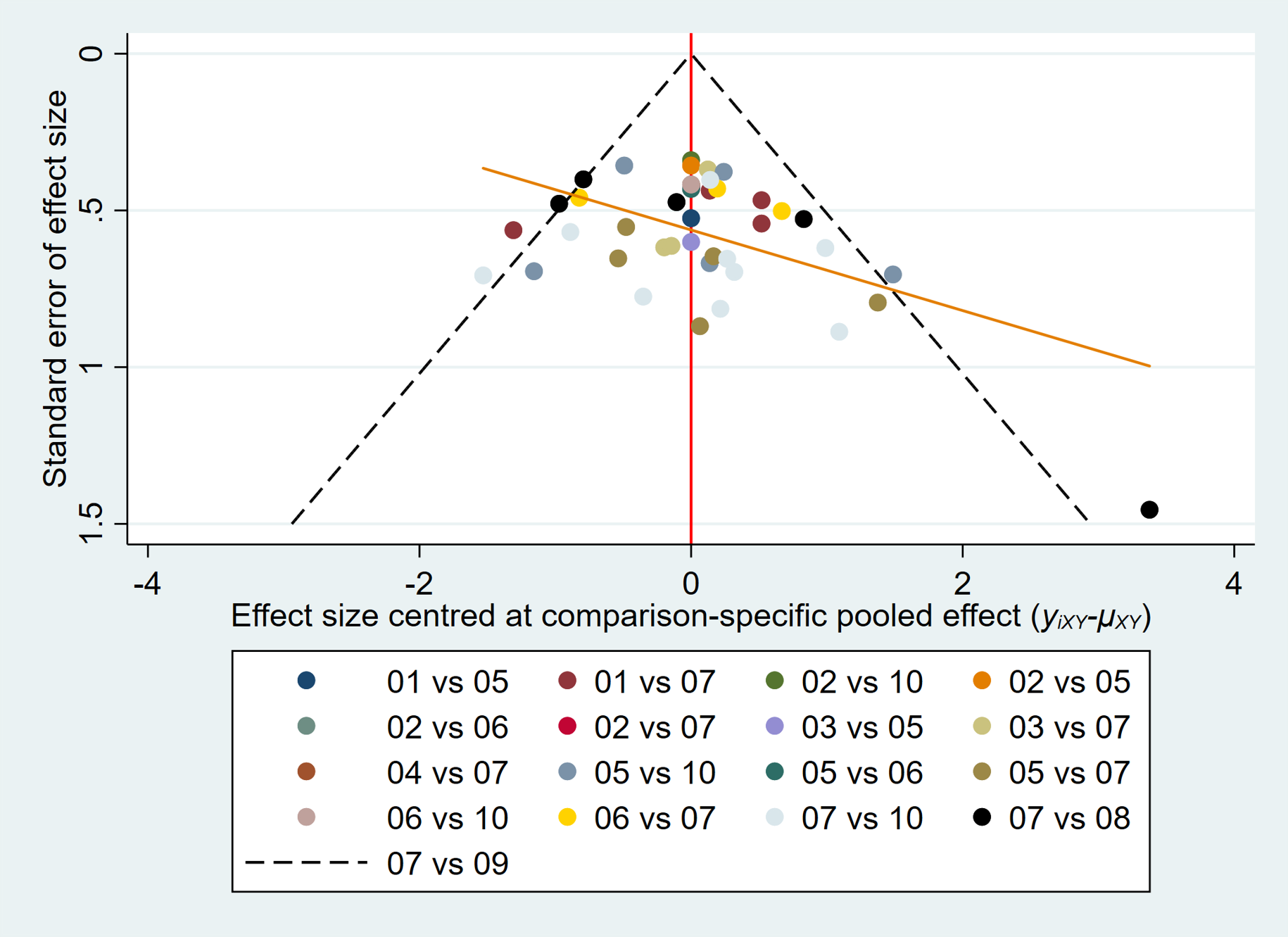


**Supplementary Figure 3.** Comparison-adjusted funnel plot. (01) Dexmedetomidine (02) Etomidate (03) Lignocaine (04) Muscle relaxant (05) Midazolam (06) NMDA-R antagonist (07) Placebo (08) Gabapentin (09) κ-R agonist (10) μ-R agonist

A.


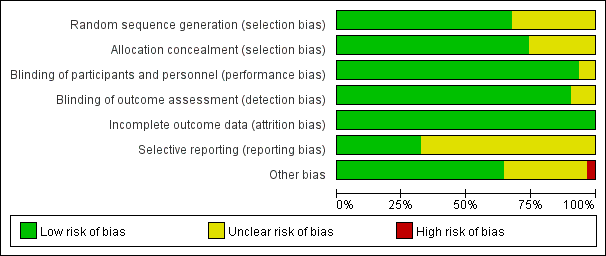


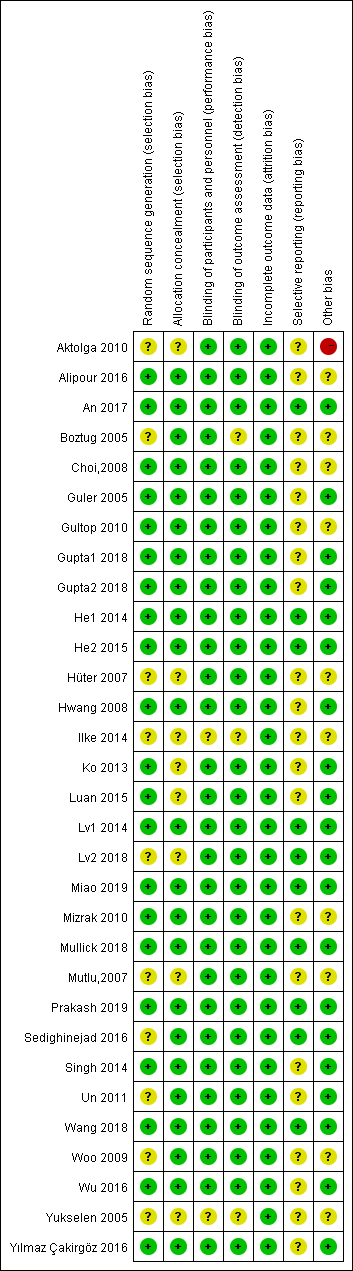
B.

**Supplementary Figure 4.** Risk of bias of the 31RCTs. (A) Risk of bias graph. (B) Risk of bias summary

| **Supplementary Table1.** | | | | | | |
| --- | --- | --- | --- | --- | --- | --- |
| **Comparison** | **Direct comparison** | | **Indirect comparison** | | **Network meta-analysis** | |
|  | **RR（95%CI）** | **Confidence** | **RR（95%CI）** | **Confidence** | **RR（95%CI）** | **Confidence** |
| B vs. P | 2.03 (1.39, 2.96) | High | Not estimable†† | — | 2.1 (0.8, 5.49) | High |
| D vs. P | 2.39 (1.48, 3.86) | Low *† | Not estimable†† | — | 2.35 (1.46, 3.85) | Low |
| P vs. E | 0.38 (0.12, 1.10) | High | 0.20 (0.084, 0.44) | Moderate | 0.25 (0.13, 0.47) | High |
| G vs. P | 2.56 (1.24, 5.25) | High | Not estimable†† | — | 2.77 (0.93, 9.38) | High |
| K vs. P | 3.12 (2.21, 4.42) | Moderate † | Not estimable†† | — | 2.94 (1.88, 4.6) | Moderate |
| L vs. P | 2.18 (1.48, 3.23) | High | Not estimable†† | — | 2.15 (1.19, 4.01) | High |
| P vs. M | 0.32 (0.19, 0.52) | Moderate † | 0.75 (0.39, 1.4) | Low | 0.46 (0.32, 0.67) | Moderate |
| U vs. P | 2.6 (1.9, 3.7) | Moderate † | 5.4 (2.9, 10.0) | Low | 3.1 (2.3, 4.3) | Moderate |
| P vs. N | 0.47 (0.26, 0.83) | Moderate † | 1.1 (0.42, 2.9) | Moderate | 0.58 (0.35, 0.95) | Moderate |
| B vs. D | — | — | 0.89 (0.3, 2.58) | Low | 0.89 (0.3, 2.58) | Low |
| B vs. E | — | — | 0.52 (0.16, 1.67) | High | 0.52 (0.16, 1.67) | High |
| B vs. G | — | — | 0.75 (0.16, 3.22) | High | 0.75 (0.16, 3.22) | High |
| B vs. K | — | — | 0.71 (0.25, 2.03) | Moderate | 0.71 (0.25, 2.03) | Moderate |
| B vs. L | — | — | 0.98 (0.31, 2.97) | High | 0.98 (0.31, 2.97) | High |
| B vs. M | — | — | 0.97 (0.34, 2.63) | Moderate | 0.97 (0.34, 2.63) | Moderate |
| B vs. U | — | — | 0.67 (0.24, 1.86) | Moderate | 0.67 (0.24, 1.86) | Moderate |
| B vs. N | — | — | 1.22 (0.41, 3.6) | Moderate | 1.22 (0.41, 3.6) | Moderate |
| D vs. E | — | — | 0.58 (0.25, 1.29) | Low | 0.58 (0.25, 1.29) | Low |
| D vs. G | — | — | 0.85 (0.23, 2.78) | Low | 0.85 (0.23, 2.78) | Low |
| D vs. K | — | — | 0.8 (0.42, 1.55) | Low | 0.8 (0.42, 1.55) | Low |
| D vs. L | — | — | 1.1 (0.51, 2.35) | Low | 1.1 (0.51, 2.35) | Low |
| M vs. D | 0.89 (0.37, 2.1) | Low *‡ | 1.0 (0.52, 2.0) | Low | 0.91 (0.52, 1.6) | Low |
| D vs. U | — | — | 0.77 (0.43, 1.32) | Low | 0.77 (0.43, 1.32) | Low |
| D vs. N | — | — | 1.37 (0.7, 2.78) | Low | 1.37 (0.7, 2.78) | Low |
| E vs. G | — | — | 1.46 (0.36, 5.38) | High | 1.46 (0.36, 5.38) | High |
| E vs. K | — | — | 1.38 (0.62, 3.06) | Moderate | 1.38 (0.62, 3.06) | Moderate |
| E vs. L | — | — | 1.88 (0.75, 4.54) | High | 1.88 (0.75, 4.54) | High |
| M vs. E | 0.48 (0.17, 1.30) | High | 0.82 (0.25, 2.60) | Moderate | 0.53 (0.27, 1.10) | High |
| U vs. E | 0.72 (0.28, 1.9) | Moderate ‡ | 1.2 (0.37, 3.7) | Moderate | 0.76 (0.39, 1.5) | Moderate |
| N vs. E | 0.23 (0.079, 0.66) | High | 0.81 (0.24, 2.70) | Moderate | 0.42 (0.20, 0.90) | High |
| G vs. K | — | — | 0.94 (0.29, 3.43) | Moderate | 0.94 (0.29, 3.43) | Moderate |
| G vs. L | — | — | 1.29 (0.36, 4.93) | High | 1.29 (0.36, 4.93) | High |
| G vs. M | — | — | 1.27 (0.4, 4.52) | Moderate | 1.27 (0.4, 4.52) | Moderate |
| G vs. U | — | — | 0.9 (0.28, 3.15) | Moderate | 0.9 (0.28, 3.15) | Moderate |
| G vs. N | — | — | 1.62 (0.49, 6.01) | Moderate | 1.62 (0.49, 6.01) | Moderate |
| K vs. L | — | — | 1.37 (0.63, 2.88) | Moderate | 1.37 (0.63, 2.88) | Moderate |
| K vs. M | — | — | 1.36 (0.76, 2.39) | Moderate | 1.36 (0.76, 2.39) | Moderate |
| K vs. U | — | — | 0.95 (0.54, 1.62) | Moderate | 0.95 (0.54, 1.62) | Moderate |
| K vs. N | — | — | 1.71 (0.88, 3.35) | Moderate | 1.71 (0.88, 3.35) | Moderate |
| M vs. L | 1.3 (0.46, 3.4) | Moderate ‡ | 0.87 (0.53, 2.1) | Moderate | 1.0 (0.53, 1.9) | Moderate |
| L vs. U | — | — | 0.7 (0.36, 1.34) | Moderate | 0.7 (0.36, 1.34) | Moderate |
| L vs. N | — | — | 1.25 (0.58, 2.75) | Moderate | 1.25 (0.58, 2.75) | Moderate |
| U vs. M | 1.7 (1.1, 2.8) | Low †‡ | 0.94 (0.48, 1.9) | Moderate | 1.4 (0.99, 2.1) | Moderate |
| N vs. M | 0.47 (0.15, 1.4) | Moderate ‡ | 1.0 (0.50, 2.1) | Moderate | 0.80 (0.44, 1.4) | Moderate |
| U vs. N | 2.8 (0.99, 8.4) | High | 1.4 (0.72, 2.8) | Moderate | 1.8 (1.0, 3.2) | High |

Approach for rating the quality of network meta-analysis (NMA) estimates. Step1. Present direct and indirect treatment estimates for each comparison of the evidence network. Step2. Rate the quality of each direct and indirect effect estimate. Step3. Present the NMA estimate. Step4. Rate quality of NMA estimate.

*Limitations (risk of bias). †Inconsistency. ‡ Imprecision. §Indirectness. ††Cannot be estimated because the drug was not connected in a loop in the evidence network.

(B) Muscle relaxant (D) Dexmedetomidine (E) etomidate (G) Gabapentin (P) Placebo (K) κ opioid receptor agonist (L) Lidocaine (M) Midazolam (U) μ opioid receptor agonist (N) NMDA receptor antagonist
