# Supplementary figures and images for: Comparison of the Effectiveness of Various Drug Interventions to Prevent Etomidate-Induced Myoclonus: A Bayesian Network Meta-Analysis
Source: Front Med (Lausanne). 2022 Apr 26;9:799156. doi: 10.3389/fmed.2022.799156 (PMC9086535; doi:10.3389/fmed.2022.799156)

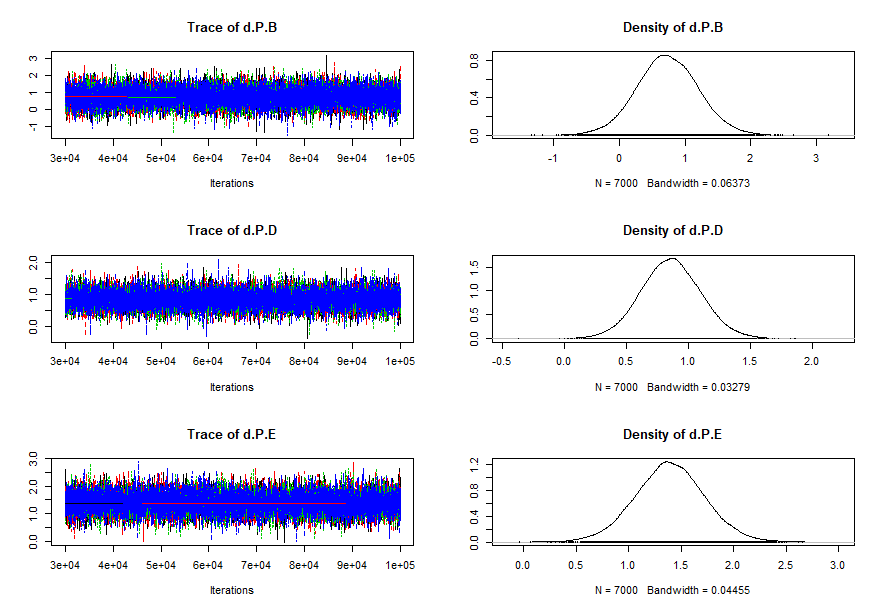

Supplement: Supplementary file 1 [file Data_Sheet_1.ZIP › Supplementary Material Presentation/Supplementary Figure 1(1).tiff]

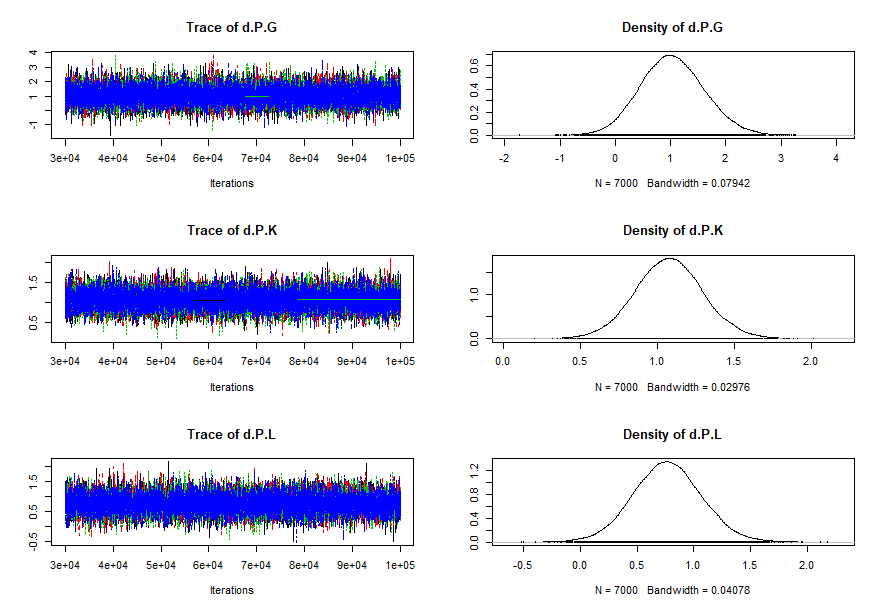

Supplement: Supplementary file 1 [file Data_Sheet_1.ZIP › Supplementary Material Presentation/Supplementary Figure 1(2)..tiff]

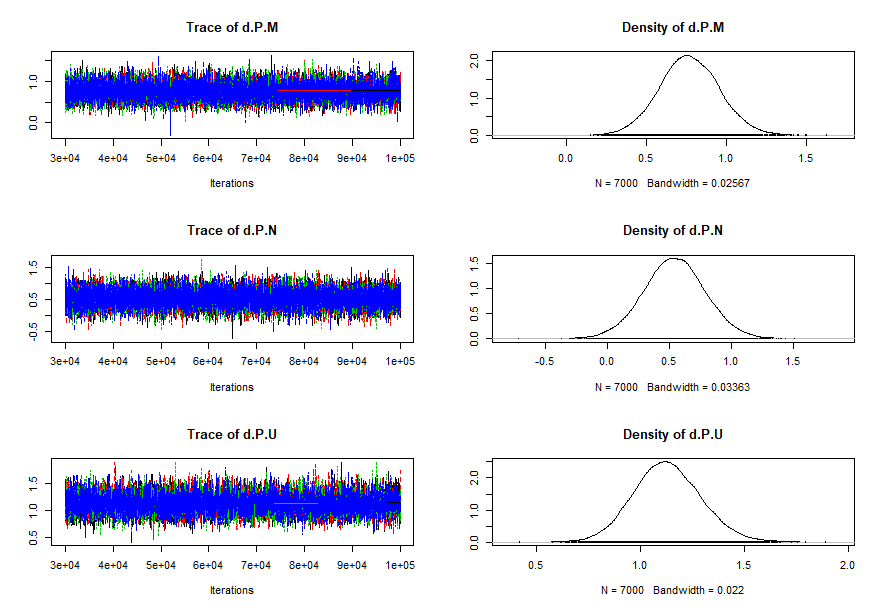

Supplement: Supplementary file 1 [file Data_Sheet_1.ZIP › Supplementary Material Presentation/Supplementary Figure 1(3)..tiff]

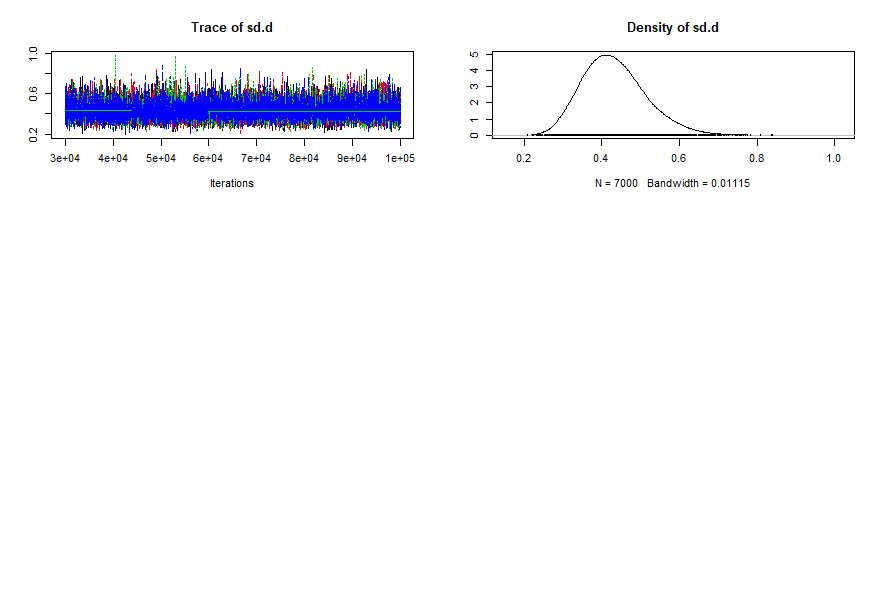

Supplement: Supplementary file 1 [file Data_Sheet_1.ZIP › Supplementary Material Presentation/Supplementary Figure 1(4)..tiff]

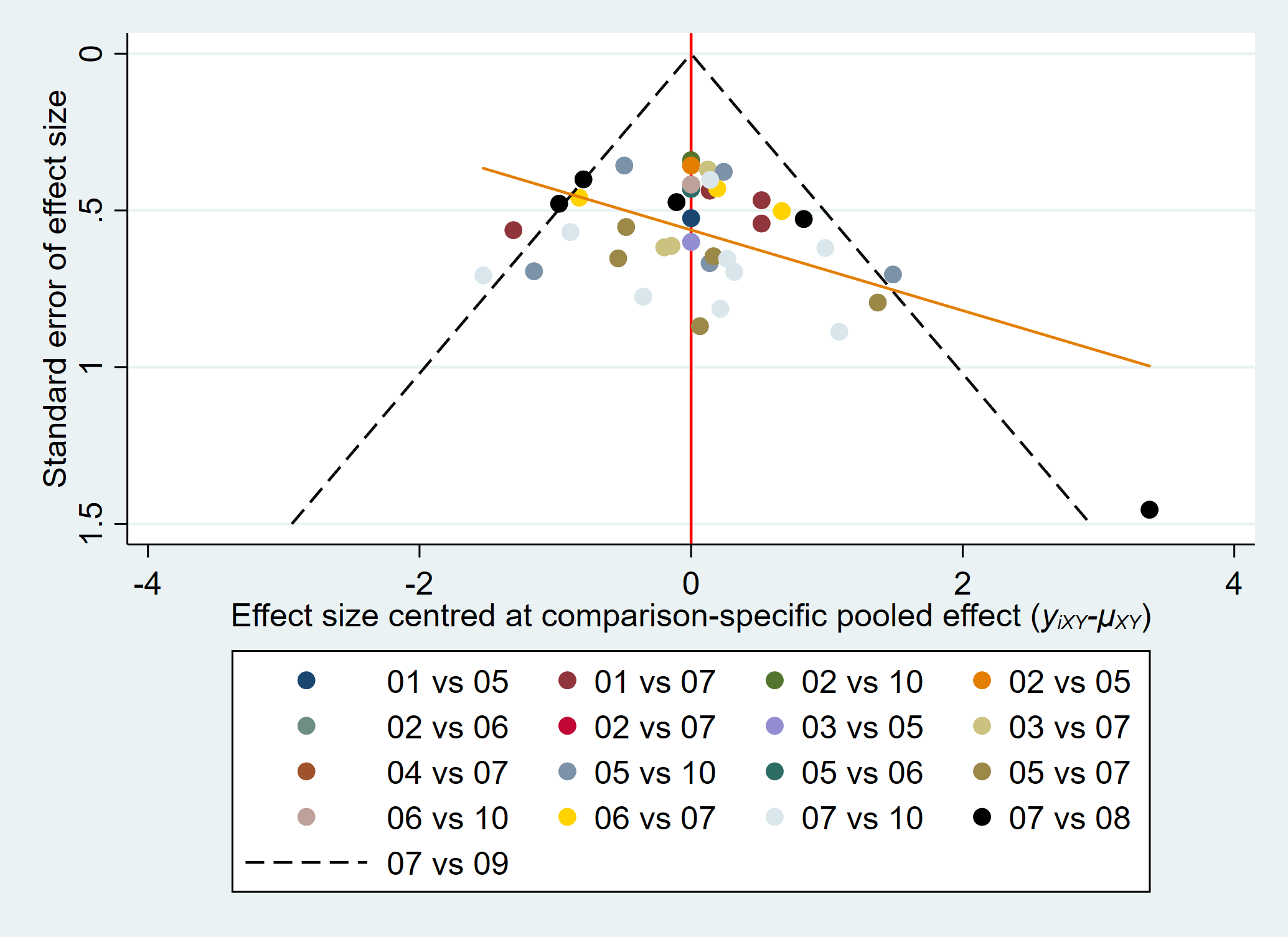

Supplement: Supplementary file 1 [file Data_Sheet_1.ZIP › Supplementary Material Presentation/Supplementary Figure 3.tif]

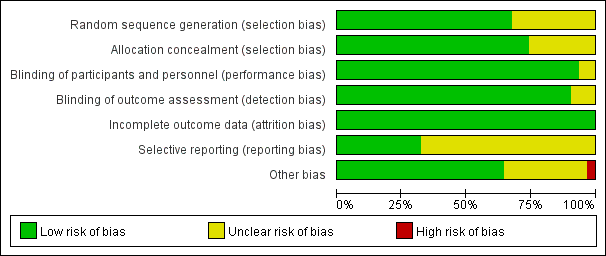

Supplement: Supplementary file 1 [file Data_Sheet_1.ZIP › Supplementary Material Presentation/Supplementary Figure 4(A).png]

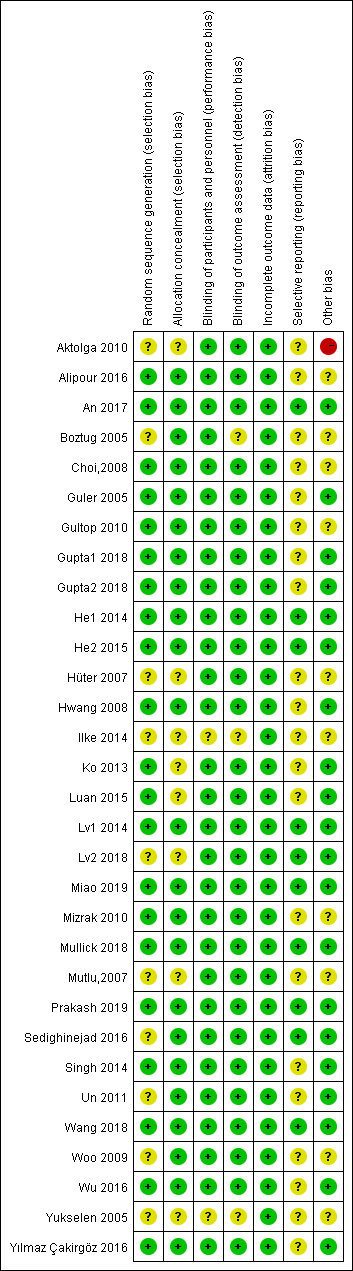

Supplement: Supplementary file 1 [file Data_Sheet_1.ZIP › Supplementary Material Presentation/Supplementary Figure 4(B).png]
